# Prognostic role of pretreatment neutrophil to lymphocyte ratio in breast cancer patients: A meta-analysis: Erratum

**DOI:** 10.1097/MD.0000000000009526

**Published:** 2017-12-29

In the article, “Prognostic role of pretreatment neutrophil to lymphocyte ratio in breast cancer patients: A meta-analysis”^[1]^, which appeared in Volume 96, Issue 45 of *Medicine*, Xiao-yi Duan's affiliation should appear as The Department of Radiology, the First Affiliated Hospital of Xi’an Jiaotong University, Xi’an, Shaanxi, P.R. China. Hang-ying Qu's affiliation should appear as The Department of Oncological Surgery, Shaanxi University of Chinese Medicine.
